# Computer-Assisted Preoperative Simulation and Augmented Reality for Plate Fixation Positioning in Genioplasty

**DOI:** 10.1007/s12663-024-02274-6

**Published:** 2024-07-31

**Authors:** Hideyuki Suenaga, Ayuko Sakakibara, Hiroshi Kawakami, Asako Taniguchi, Kazuto Hoshi

**Affiliations:** Department of Oral-Maxillofacial Surgery and Orthodontics, The University of Tokyo Hospital, 7-3-1 Hongo, Bunkyo-Ku, Tokyo, 113-8655 Japan

**Keywords:** Genioplasty, Augmented reality, Plate fixation, Computer simulation

In orthognathic surgery, Computed tomography (CT) images are used for three-dimensional (3D) planning. Simulating the surgical procedure makes the surgery easier. The surgeon can access the 3D images using this technique which facilitates bone segments positioning that is required to fix the plate [1]. Using 3D printing, patient-specific cutting guides [2], and patient-specific implants [3]. for genioplasty could be a beneficial aid. Augmented reality (AR), a new imaging technology that combines virtual and real-world images into a single environment, has recently been introduced to assist oral surgeons in visualising sites that cannot be seen directly. In order to locate the plate fixation in genioplasty, the authors developed a treatment procedure using a CT-based computer-assisted design and an intraoperative AR-guided system [4].

The Paulus chin plate of 6 mm (Lorenz Plating System, Biomet Microfixation, Jacksonville, FL, USA) and the 3D scanner were used to scan the teeth (TRIOS3, 3Shape, Copenhagen, Denmark). A 320-row area detector CT scanner was used to scan the face (Aquilion ONE ViSION, Canon Medical System, Tochigi, Japan), which acquired CT scans (0.35 mm/pixel size and 0.5 mm/pixel slice thickness). Mimics software was used in conjunction with a 3D scanner and CT data to recreate 3D surfaces (version 24.0, Materialise, Leuven, Belgium). A genioplasty simulation was performed using the 3D mandible model, according to the surgical plan. Mimics software was used to calculate bone movement and screw positions (Fig. 1). In the actual AR surgery (Fig. 2, Supplementary video), a 3840 × 2160 pixels image resolution 4 K camera was used (Next Vision, Yoshida, Tokyo, Japan). Prior to distortion removal, the camera was calibrated to obtain the intrinsic matrix K and lens distortion coefficients. C +  + was used to implement all of the core algorithms [4]. The medical ethics committee of the Graduate School of Medicine, affiliated to the University of Tokyo in Japan, provided due approval for this study. During the real operation (Fig. 3 and 4), the positioning of plate in genioplasty was done based on this preoperative planning and AR. It is anticipated that this simulation method will help surgeons in planning and performing operative procedures more precisely in orthognathic surgery. With the assistance of see-through AR guidance, our intraoperative markerless video system is capable of addressing issues pertaining to initial alignment and maintaining the accuracy of registration in real-time, eliminating the need for manual adjustment. Our work is presumably the first to achieve marker-less video see-through AR for genioplasty. This method is anticipated to be incorporated increasingly into orthognathic surgery to help surgeons in executing operative procedures with utmost precision. This AR system can be easily applied in the surgical field without conventional navigation-guided systems or 3D-printed surgical guides.

**Fig. 1:**
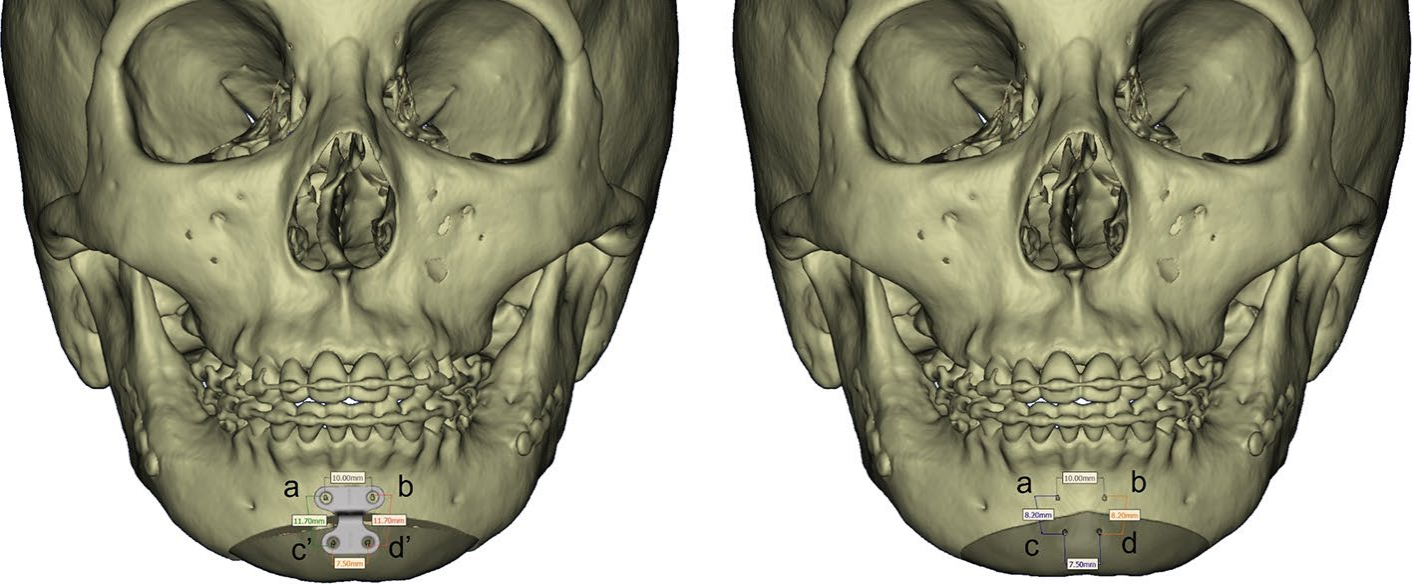
Pre-surgery plan and simulation with the virtual model of chin plate 6 mm7. (left) Frontal view following bone movement; (right) front view before bone movement. The distance between the points before and after bone movement: a, b, c, d are positions before bone movement; c’, d’ are positions after bone movement. a ~ b = 10.0 mm; c ~ d = 7.5 mm; a ~ c = 8.2 mm; b ~ d = 8.2 mm; c’ ~ d’ = 7.5 mm; a ~ c’ = 11.7 mm; b ~ d’ = 11.7 mm

**Fig. 2:**
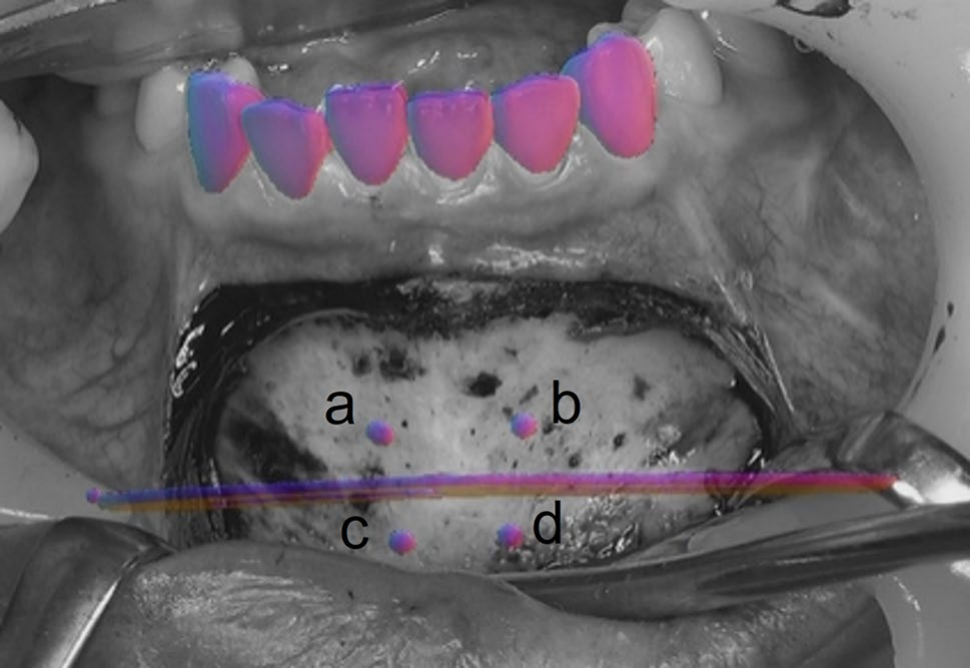
Positioning of plate and screw during AR navigation-guided surgery

**Fig. 3:**
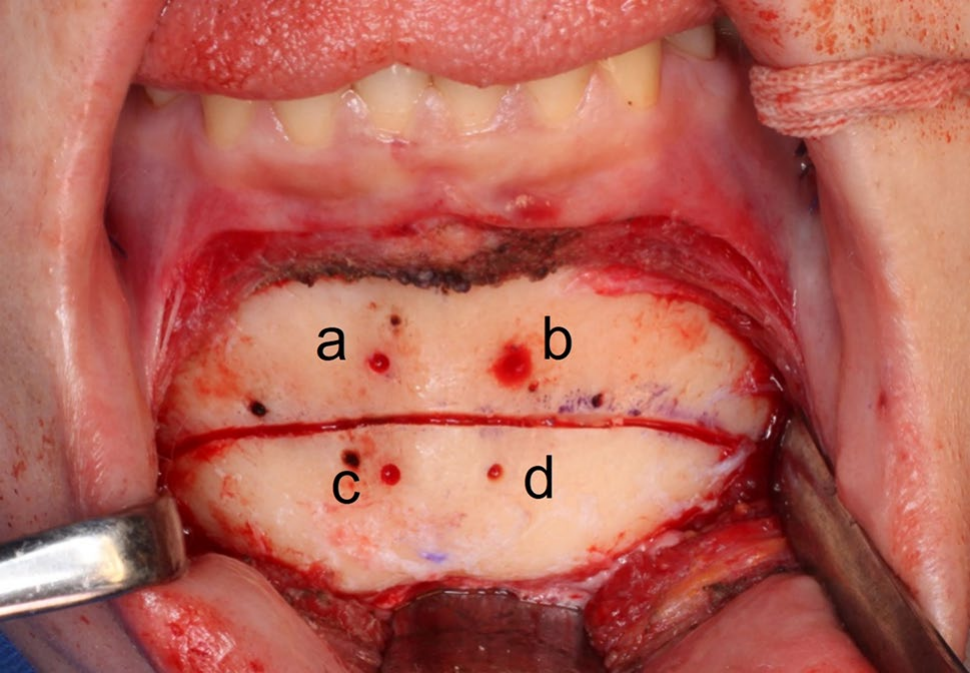
Positioning of plate and screw during actual surgery. Front view before the bone movement

**Fig. 4:**
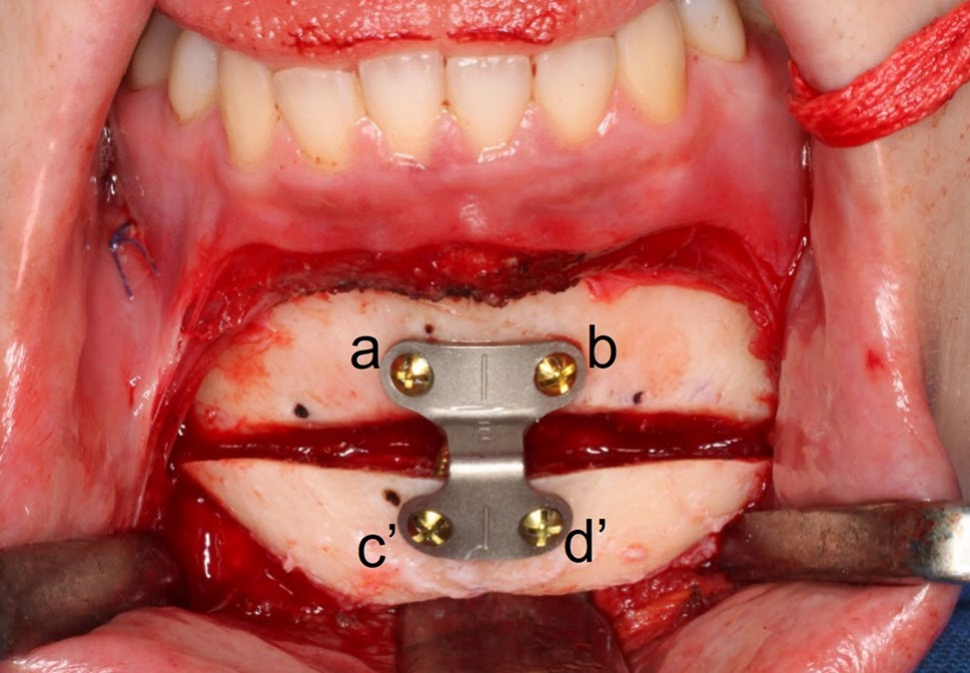
Positioning of plate and screw during actual surgery. Front view following bone movement

## Supplementary Information

Below is the link to the electronic supplementary material.

Supplementary file 1 (MP4 37036 kb)

## Data Availability

All data generated during this study are included in this article and its supplementary file.
